# Does exercise environment moderate the effects of Tai Chi on depression and anxiety? A systematic review and meta-analysis of indoor vs. outdoor practice settings

**DOI:** 10.3389/fspor.2026.1955833

**Published:** 2026-09-29

**Authors:** Nan Zhang, Guidan Hu, Xiujie Ma

**Affiliations:** 1 School of Wushu, Chengdu Sport University, Chengdu, China; 2 Chinese GuoShu Academy, Chengdu Sport University, Chengdu, China

**Keywords:** Tai Chi, depression, anxiety, exercise environment, indoor and outdoor practice, systematic review, meta-analysis

## Abstract

**Background:**

Depression and anxiety are highly prevalent mental disorders that impose a substantial global health burden. As a traditional mind–body exercise, Tai Chi has demonstrated well-established non-pharmacological benefits for alleviating negative emotional symptoms. However, previous meta-analyses have primarily focused on intervention dosage (e.g., frequency and duration) or participant characteristics, while largely overlooking the potential influence of the physical exercise environment as a contextual ecological factor. This systematic review and meta-analysis aimed to evaluate the overall effects of Tai Chi on depression and anxiety and to determine whether indoor and outdoor practice settings moderate these mental health benefits.

**Methods:**

This systematic review and meta-analysis was conducted in accordance with the PRISMA guidelines. A comprehensive literature search was performed in CNKI, Web of Science, PubMed, the Cochrane Library, and Embase for studies published from database inception to June 2026. Randomized controlled trials (RCTs) investigating Tai Chi as the primary intervention and reporting outcomes related to depression and/or anxiety were included. Two reviewers independently screened studies, extracted data, and assessed the risk of bias using the RoB 2.0 tool. The certainty of evidence was evaluated using the GRADE approach. Standardized mean differences (SMDs) with 95% confidence intervals (CIs) were pooled using a random-effects model. Heterogeneity was assessed using Cochran's *Q* test and the *I*^2^ statistic. Subgroup analyses and meta-regression were performed to examine the potential moderating effect of the exercise environment.

**Results:**

A total of 15 RCTs involving 847 participants were included. Tai Chi significantly reduced both depressive symptoms (SMD = −0.93, 95% CI: −1.25 to −0.61, *I*^2^ = 75.1%) and anxiety symptoms (SMD = −0.93, 95% CI: −1.23 to −0.64, *I*^2^ = 62.9%). Although univariate subgroup analyses descriptively showed larger effect sizes in outdoor settings than in indoor settings for both depression (SMD = −1.06 vs. −0.88) and anxiety (SMD = −1.08 vs. −0.90), outdoor subgroups comprised few studies (*k* = 4) with substantial heterogeneity (*I*^2^ > 78%). Crucially, multivariable meta-regression analyses confirmed that the exercise environment was not a statistically significant independent moderator for either depression (*p* = 0.90) or anxiety (*p* = 0.21) after adjusting for age, intervention duration, and dosage parameters.

**Conclusions:**

Tai Chi is an effective non-pharmacological intervention for alleviating depression and anxiety. However, current empirical evidence does not support an independent moderating effect of the exercise environment. The observed numerical differences between indoor and outdoor settings should be interpreted cautiously as preliminary and hypothesis-generating rather than confirmatory. Well-designed RCTs directly comparing standardized indoor vs. outdoor practice environments are warranted.

## 1 Introduction

Depression and anxiety are among the most prevalent mental disorders worldwide and represent a substantial public health burden. According to the World Health Organization (WHO), more than 320 million people worldwide are affected by depression and over 260 million experience anxiety disorders, accounting for 4.4% and 3.6% of the global population, respectively (1). Depression and anxiety frequently co-occur, substantially impairing quality of life and imposing considerable socioeconomic costs (2, 3). Globally, these disorders are estimated to result in nearly US$1 trillion in lost productivity each year (4). Pharmacotherapy, particularly selective serotonin reuptake inhibitors (SSRIs), and psychotherapy remain the primary treatment approaches for these conditions. Although both have demonstrated substantial efficacy in symptom management, SSRIs may cause adverse effects, including drowsiness, weight gain, and sexual dysfunction (5, 6), and prolonged use may be associated with difficulties in discontinuation and withdrawal symptoms (7, 8). Likewise, the widespread implementation of cognitive behavioral therapy (CBT) continues to be constrained by limited availability of trained therapists, restricted access to mental health services, and challenges related to patient adherence (9, 10). Consequently, identifying safe, effective, and accessible non-pharmacological interventions has become an important research priority in the field of mental health.

Tai Chi, a traditional Chinese mind–body exercise integrating gentle physical movements, breath regulation, and mental focus (11), has gained widespread application in modern health promotion and clinical rehabilitation. Over its prolonged historical evolution and lineage transmission, Tai Chi has developed diverse styles and adapted formats that differ markedly in movement complexity, practice tempo, and physiological load. For example, Yang-style Tai Chi is characterized primarily by expansive, graceful, gentle, slow, and continuous movements performed at an even tempo, making it widely utilized in health promotion and clinical rehabilitation research (12). In contrast, Chen-style Tai Chi combines softness with hardness, alternates between fast and slow pacing, and incorporates explosive force (*fajin*) and low-stance footwork, thereby imposing comparatively higher demands on lower-limb muscular strength and neuromuscular coordination (13, 14). Furthermore, modern simplified routines (e.g., 24-form, 16-form, and 8-form) and chair-based adaptations designed for special populations feature relatively straightforward and accessible movements that further lower the physical activity threshold, facilitating practice among frail older adults or mobility-impaired individuals (15, 16). A substantial body of randomized controlled trials and systematic reviews has demonstrated that regular Tai Chi practice significantly alleviates negative emotional symptoms such as depression, anxiety, and psychological stress, while enhancing overall mental health (17–21). Nevertheless, the magnitude of intervention effects varies across studies. To explain this heterogeneity, previous systematic reviews and meta-analyses have primarily focused on intervention dosage (e.g., training frequency, session duration, and intervention period), participant characteristics (e.g., age, sex, and health status) (22, 23), and different Tai Chi styles, while paying relatively limited attention to the physical context in which the exercise is delivered.

Indeed, the physical environment is not merely a passive backdrop for intervention delivery, but an active contextual factor that shapes exercise experiences, physiological responses, and psychological restoration. In recent years, growing evidence suggests that the health benefits of exercise interventions depend not only on traditional FITT components (frequency, intensity, time, and type), but are also moderated by external contextual factors such as the exercise setting, where different physical environments can alter emotional experiences during exercise and thereby influence intervention efficacy (24–26) For Tai Chi, the exercise environment carries unique historical and cultural resonance: deeply rooted in traditional Chinese philosophical concepts of *Tian Ren He Yi* (the harmony and unity between humanity and nature) and living in accord with natural rhythms, Tai Chi was originally conceived not only as a martial art, but as a holistic cultivation system wherein practitioners attune breath and bodily rhythms to natural surroundings, making the outdoor natural setting its historical and ecological baseline (27, 28). Concurrently, as Tai Chi has been widely integrated into modern healthcare and rehabilitation, practice settings have gradually expanded into indoor environments-such as hospital wards, rehabilitation centers, and community rooms-largely to accommodate the physical needs of specific populations and mitigate weather-related constraints. However, existing research on environmental effects remains predominantly confined to general physical activities (29, 30), leaving mind–body exercises like Tai Chi largely underexplored. Although previous randomized controlled trials have implemented Tai Chi across diverse indoor and outdoor venues (e.g., hospitals, community centers, urban parks, and green spaces), the majority have treated practice settings merely as implementation sites rather than intentionally designing or analyzing them as potential effect modifiers. Given that Tai Chi places central emphasis on bodily awareness, respiratory regulation, and mindful focus, whether this spatial transition from traditional outdoor settings to modern indoor environments alters its antidepressant and anxiolytic efficacy remains without systematic quantitative evidence. Consequently, whether the exercise environment moderates the mental health benefits of Tai Chi represents a critical scientific question encompassing both cultural significance and clinical translational value.

Therefore, this study conducted a systematic review and meta-analysis to evaluate the overall effects of Tai Chi on depression and anxiety. In addition, subgroup analysis based on practice setting (indoor vs. outdoor) were performed to investigate whether the exercise environment moderates the mental health benefits of Tai Chi. The findings are expected to provide evidence to inform the optimization of Tai Chi intervention design and the implementation of evidence-based strategies for mental health promotion.

## 2 Materials and methods

This systematic review and meta-analysis was designed and conducted in accordance with the methodological recommendations of the Cochrane Handbook for Systematic Reviews of Interventions. The reporting of this study followed the Preferred Reporting Items for Systematic Reviews and Meta-Analyses (PRISMA 2020) statement (31) to ensure transparency and completeness throughout the review process. The review protocol was prospectively registered with the International Prospective Register of Systematic Reviews (PROSPERO; registration No. CRD420261440793). In addition, the certainty of evidence for all primary outcomes was assessed using the Grading of Recommendations Assessment, Development and Evaluation (GRADE) approach.

### 2.1 Search strategy

A comprehensive literature search was conducted in Web of Science, PubMed, the Cochrane Library, Embase, and China National Knowledge Infrastructure (CNKI) to identify RCTs investigating the effects of Tai Chi on mental health benefits. The search covered all eligible studies published from database inception to June 2026, with no language restrictions. A combination of controlled vocabulary [e.g., Medical Subject Headings (MeSH)] and free-text terms was used, and search terms were combined using the Boolean operators AND and OR.

### 2.2 Inclusion and exclusion criteria

The eligibility criteria were established according to the PICOS framework (Participants, Intervention, Comparator, Outcomes, and Study design).

Studies were included if they met the following criteria: (1) study design: randomized controlled trials (RCTs) reported in either peer-reviewed journal articles or graduate theses/dissertations. For multi-arm trials, data had to allow extraction of an independent comparison between a Tai Chi intervention group and an eligible control group; (2) participants: individuals with clearly reported baseline characteristics, regardless of age, sex, or health status; (3) intervention: Tai Chi, including traditional or modified forms (e.g., Yang-style, Chen-style, or the 24-form Tai Chi), served as the sole or primary exercise intervention; (4) comparator: studies included a clearly defined control group, such as a usual-care control, wait-list control, no-intervention control, or conventional exercise/routine care control that did not involve environmental manipulation; (5) outcomes: depression and/or anxiety were assessed using validated quantitative instruments, including depression scales (e.g., SDS, BDI, PHQ-9, HAMD, GDS-15, and CES-D) and anxiety scales (e.g., SAS, GAD-7, HAMA, and STAI); (6) data availability: studies reported sufficient pre- and post-intervention data or adequate statistical information (e.g., means, standard deviations, and sample sizes) to calculate effect sizes; and (7) practice setting: studies explicitly reported, or provided sufficient information to determine, a single practice setting. Indoor practice settings included gymnasiums, community activity centers, rehabilitation facilities, and home-based settings, whereas outdoor practice settings included parks, community green spaces, playgrounds, and other natural or semi-natural open environments.

Studies were excluded if they met any of the following criteria: (1) non-randomized studies, including quasi-experimental studies, non-randomized controlled studies, single-group pre-post studies, case reports, qualitative studies, or systematic reviews; (2) studies that did not clearly report the practice setting, used mixed indoor–outdoor settings without separately extractable data, or employed simulated outdoor practice settings (e.g., virtual reality) rather than actual environmental exposure; (3) multimodal interventions in which Tai Chi was combined with other forms of exercise (e.g., jogging or resistance training) or psychological/behavioral therapies (e.g., cognitive behavioral therapy or meditation) and the independent effects of Tai Chi could not be isolated; (4) studies reporting only biomechanical outcomes, physical fitness measures (e.g., upper-limb strength, balance, gait characteristics, or joint flexibility), physiological or biochemical indicators (e.g., heart rate variability, salivary cortisol, or electroencephalographic activity), or disease-specific functional outcomes, without reporting any mental health benefits or providing sufficient quantitative data for analysis; and (5) study protocols, conference abstracts, case reports, systematic reviews, industry reports, or secondary analyses based on RCT datasets that had already been included in another eligible publication, in order to avoid duplicate data.

Two reviewers independently screened the titles and abstracts of all retrieved records, removed duplicate publications, and assessed the full texts of potentially eligible studies according to the predefined inclusion and exclusion criteria. Any disagreements were resolved through discussion, and when consensus could not be reached, a third reviewer was consulted to make the final decision.

### 2.3 Data extraction

Study selection and data extraction were performed independently by two reviewers. Any disagreements were resolved through discussion with a third reviewer. When numerical data were incomplete or presented only in graphical form, the corresponding authors were contacted by email to obtain the required information. The following data were extracted from each eligible study: (1) study characteristics (e.g., first author, publication year, country, and sample size); (2) participant characteristics (e.g., age and sex); (3) details of the intervention (e.g., program name, duration, weekly frequency, duration of each session); (4) outcome assessment instruments used to evaluate mental health; and (5) outcome measures.

### 2.4 Quality assessment

Data related to participants, interventions, and outcome measures were independently extracted by two reviewers using a standardized data extraction form. The risk of bias for each included study was assessed independently using the Cochrane Risk of Bias 2.0 (RoB 2.0) tool. This instrument evaluates five domains of potential bias: (1) bias arising from the randomization process; (2) bias due to deviations from the intended interventions; (3) bias due to missing outcome data; (4) bias in the measurement of the outcome; and (5) bias in the selection of the reported result. In accordance with the Cochrane Handbook for Systematic Reviews of Interventions, each domain and the overall risk of bias were judged as “low risk,” “some concerns,” or “high risk.” Any disagreements during the assessment process were resolved through discussion with a third reviewer.

### 2.5 Statistical analysis

The statistical analysis followed the methodological framework recommended by the Cochrane Handbook for Systematic Reviews of Interventions. All data pooling, heterogeneity testing, forest plot generation, and publication bias analyses were performed using Stata 17.0. The methodological quality of the included studies was assessed using the Cochrane RoB 2.0 tool for risk of bias assessment. Since the mental health outcome measures in the included studies were all continuous variables and employed diverse measurement instruments, the standardized mean difference (SMD) with its 95% confidence interval (95% CI) was adopted as the composite effect size metric. The SMD was calculated as the difference in means between the intervention and control groups divided by the pooled standard deviation. Given the relatively small sample sizes in some included studies, Hedges' *g* correction was applied to the SMD where appropriate to reduce small-sample bias. Data extraction preferentially used post-intervention means and standard deviations; for data reported as medians and interquartile ranges, these were converted to means and standard deviations using the method proposed by Wan et al. (32), and the direction of effect sizes was uniformly adjusted for scales with inconsistent scoring directions. Between-study heterogeneity was assessed using the Cochrane Q test and the *I*^2^ statistic. A significant Q test value may indicate the presence of heterogeneity, while the *I*^2^ value reflects the degree of heterogeneity-with values of approximately 25%, 50%, and 75% interpreted as low, moderate, and high heterogeneity, respectively (33, 34). Given the clinical and methodological heterogeneity across the included studies in terms of population characteristics, intervention protocols, and outcome measurement tools, a random-effects model was prespecified for effect size pooling. This approach is considered more conservative, as it adequately accounts for potential differences in true effect sizes across studies. Publication bias was evaluated using Begg's rank correlation test and Egger's linear regression test, supplemented by visual inspection of funnel plots. In addition, sensitivity analyses were conducted using the leave-one-out method to identify individual studies that might unduly influence the pooled effect estimates. As a supplementary sensitivity analysis addressing potential differences in publication and peer-review status, the primary meta-analyses were repeated after excluding graduate theses/dissertations and retaining only studies published in peer-reviewed journals. The resulting pooled effect estimates were compared with those obtained from all eligible studies to evaluate the robustness of the main findings.

## 3 Results

### 3.1 Study selection

A systematic search of Web of Science, PubMed, the Cochrane Library, Embase, and China National Knowledge Infrastructure (CNKI) identified 5,974 records published up to 20 June 2026. After removing 1,749 duplicate records and excluding 4,152 records based on title and abstract screening, 73 articles underwent full-text assessment for eligibility. Of these, 58 studies were excluded for the following reasons: inappropriate intervention or study focus (*n* = 24), insufficient outcome data (*n* = 17), and mixed indoor–outdoor practice settings that did not permit separate data extraction (*n* = 17). Consequently, 15 studies met the eligibility criteria and were included in the final meta-analysis, as illustrated in the PRISMA flow diagram (Figure 1). The characteristics of the included studies are summarized in Table 1.

**Figure 1:**
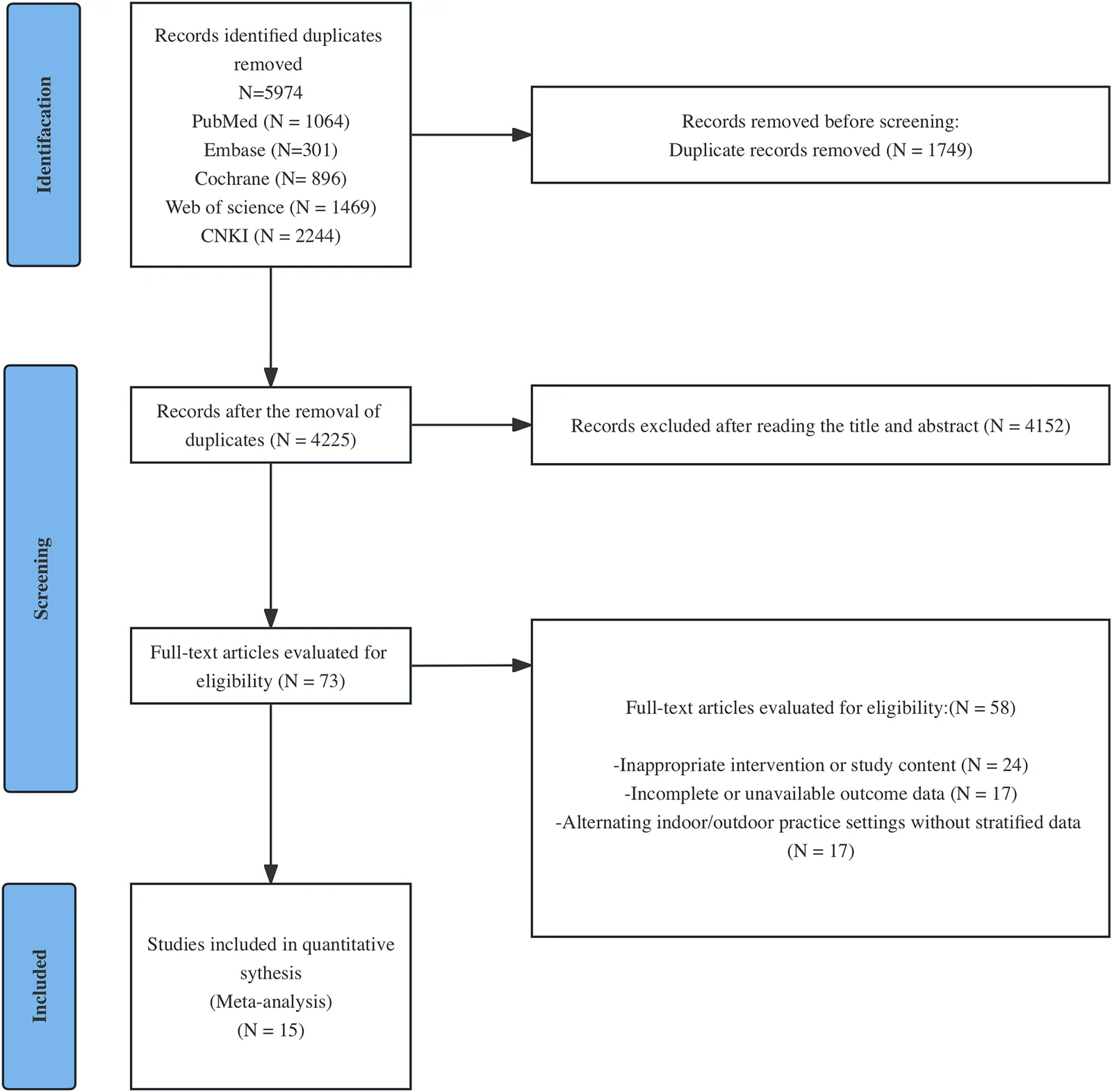
Flow of study selection.

**Table 1:** Included trial characteristics.

Trial ID	Country	Participants	*N* (total)	Age Mean (Range)	Gender, % Female	T	C	Outcome Measures	Research Type
Cui JL (2020)	China	University students with subclinical depression	26	18–25	50	13	13	BDI-II	RCT
Y Y Fang (2021)	China	Elderly adults with insomnia	63	≥85	NR	29	34	PSQI; SAS	RCT
He YX (2024)	China	Elderly inpatients with MCI	36	≥60	61.1	18	18	PSQI; HADS	RCT
Qin QC (2023)	China	Nursing home elderly	40	60–74	NR	20	20	GDS-15; SAS; GWB	RCT
Zhang ZP (2024)	China	Postoperative breast cancer patients	20	30–70	100	10	10	DASS-21; FACT-B	RCT
Zhou W. et al. (2022)	China	Nasopharyngeal carcinoma patients undergoing radiotherapy	85	≥18	41.2	42	43	POMS-SF	RCT
Li, F. et Al. (2001)	USA	Low-active older adults	72	≥65	76.5	40	32	CES-D; PANAS	RCT
Song, J. et al. (2022)	China	Elderly women with knee OA	35	60–75	100	18	17	SF-36 MCS; PSQI; SAS; SDS	RCT
Taylor-Piliae, R.E., et al. (2014)	USA	Community stroke survivors	101	≥50	43.6	53	48	CES-D; PSQI; SF-36 MCS	RCT
Wu, J.S. et al. (2023)	China	Young adults with subthreshold depression	103	18–25	68.0	49	54	PHQ-9; GAD-7; SF-36	RCT
Zhang YG (2024)	China	Primary Sjögren's syndrome patients	20	40–60	90	10	10	HADS; SF-36	RCT
Cheng L et al. (2021)	China	Elderly adults	39	≥60	100	19	20	POMS; PSQI	RCT
Song QH. et al. (2014)	China	Elderly adults with anxiety	32	60–75	NR	16	16	HAMA; GQOLI-74	RCT
Yang XW (2018)	China	Elderly with primary hypertension	75	60–75	54.7	37	38	PSQI; AIS; SAS; GDS	RCT
Deng HM. et al. (2013)	China	Community elderly residents	100	60–85	57	50	50	SAS; SDS; PSQI	RCT

BDI-II, Beck Depression Inventory-II; PSQI, Pittsburgh Sleep Quality Index; SAS, Self-Rating Anxiety Scale; HADS, Hospital Anxiety and Depression Scale; GDS-15, Geriatric Depression Scale-15; GWB, General Well-Being Schedule; DASS-21, Depression Anxiety Stress Scales-21; FACT-B, Functional Assessment of Cancer Therapy-Breast; POMS-SF, Profile of Mood States-Short Form; CES-D, Center for Epidemiologic Studies Depression Scale; PANAS, Positive and Negative Affect Schedule; SF-36 MCS, Short Form-36 Mental Component Summary; SDS, Self-Rating Depression Scale; PHQ-9, Patient Health Questionnaire-9; GAD-7, Generalized Anxiety Disorder-7; AIS, Athens Insomnia Scale; GQOLI-74, Generic Quality of Life Inventory-74; HAMA, Hamilton Anxiety Rating Scale; NR, not reported; RCT, randomized controlled trial.

### 3.2 Characteristics of eligible studies

A total of 15 RCTs were included in this systematic review, comprising 8 studies published as peer-reviewed journal articles (35–42) and 7 graduate theses/dissertations (43–49). Nine studies were reported in Chinese and six in English. These studies were conducted in China (13 studies) (35, 36, 38, 39, 41–49) and the United States (2 studies) (37, 40). Sample sizes across studies ranged from 20 to 103 participants, comprising a total of 847 subjects, with ages ranging from 18 to 85 years (some studies only reported ≥60 or ≥85 years). All included studies used negative emotions such as depression and anxiety as core outcome measures; among them, 3 studies additionally included sleep quality as a secondary outcome (35, 45, 48), while the remaining 12 focused solely on depression- and anxiety-related emotional indicators (36–44, 46, 47, 49).

### 3.3 Interventions and controls

The characteristics of the Tai Chi interventions are summarized in Table 2. Across the included studies, Tai Chi was performed in either indoor practice settings or outdoor practice settings. Indoor settings included nursing homes, community activity centers, hospital wards, and fitness centers, whereas outdoor settings comprised public recreational spaces such as residential squares and hospital parks. The Tai Chi interventions varied in style and format, although the simplified 24-form Yang-style Tai Chi was the most commonly used protocol. Specifically, eight studies employed the standard simplified 24-form Tai Chi or a simplified Yang-style version (35, 37, 40–42, 44–46). Three studies used modified protocols derived from the 24-form, including the 8-form Tai Chi (47), modified 8-form Tai Chi (38), and a self-developed seated Tai Chi program (48). The remaining studies adopted other forms, including the 16-form Tai Chi (43), 8-form Tai Chi Qigong (49), the 18-form Chen-style Tai Chi Essence (39), and music-accompanied Tai Chi (36). The intervention duration ranged from 5 to 24 weeks, with most studies implementing a 12-week intervention (36, 38, 40, 41, 43, 44, 46–49). The shortest intervention lasted 5 weeks (42), whereas the longest lasted 24 weeks (35, 37). Training frequency was most commonly three sessions per week (35, 38, 40, 41, 43–48), although several studies adopted higher training frequencies, including five sessions per week (42) or one to two sessions per day (36, 39, 45). Session duration ranged from 15 to 90 min, with 60-min sessions being the most frequently adopted (35, 37, 38, 40, 41, 44, 46, 48, 49). Control conditions were predominantly non-exercise or usual-care comparators. Fourteen studies employed inactive or routine control conditions, including no-intervention controls (43, 44, 46, 48), wait-list controls (37, 41), usual clinical care (40, 42, 47), health education (38), usual treatment combined with medication (39, 49), usual daily activities without additional structured exercise (35), and walking (36). One study used an active control, in which participants performed brisk walking matched for exercise volume to control for the non-specific effects of physical activity (45). Across all included studies, Tai Chi interventions were delivered as supervised group-based structured training programs, with standardized training frequency and session duration to ensure consistency of the intervention dose. No study implemented a high-intensity exercise protocol.

**Table 2:** Characteristics of included trials.

Trial ID	Intervention content	Intervention site	Setting	Duration	Session (min)	Sessions per Week	Control Arm
Cui JL (2020)	16-form Tai Chi (Yang style)	Indoor (Fixed indoor training hall)	S, Q	12 weeks	90 min	3 times/week	Blank control (Maintain usual lifestyle)
Y Y Fang (2021)	Seated Tai Chi (24-form adapted)	Indoor (Nursing home dance room)	S, Q	12 weeks	60 min	3 times/week	Blank control (Maintain usual lifestyle)
He YX (2024)	8-form Tai Chi (Fuzhong Li version)	Indoor (Institution indoor activity hall)	S, Q	12 weeks	30 min	3 times/week	Routine medical care & regular daily activities
Qin QC (2023)	24-form simplified Tai Chi	Indoor (Functional classroom)	S, Q	12 weeks	60 min	3 times/week	Blank control (Maintain regular lifestyle)
Zhang ZP (2024)	8-form Tai Chi exercise	Indoor (Traditional Chinese Medical clinic)	S, Q	12 weeks	60 min	2 times/week	Routine therapy & moxibustion (No regular exercise)
Zhou W. et al. (2022)	24-form Tai Chi (video-guided)	Indoor (Clinical ward activity room)	S → U, I	5 weeks	15–30 min	5 times/week	Routine care (Radiotherapy health education)
Li, F. et al. (2001)	Yang-style simplified Tai Chi	Indoor (Community senior/retirement center)	S, Q	24 weeks	60 min	2 times/week	Waitlist control (Maintain routine daily activities)
Song, J. et al. (2022)	Modified 8-form Tai Chi	Indoor (Community center group facility)	S, Q	12 weeks	60 min	3 times/week	Health education & home-based exercise guidance
Taylor-Piliae, R.E., et al (2014)	Yang-style 24-form Tai Chi	Indoor (Outpatient rehabilitation center)	S, Q	12 weeks	60 min	3 times/week	Routine care (Written materials & weekly calls)
Wu, J.S. et al (2023)	24-form simplified Tai Chi	Indoor (University gymnasium center)	S, Q	12 weeks	60 min	3 times/week	Waitlist control (Maintain routine lifestyle)
Zhang YG (2024)	24-form simplified Tai Chi	Outdoor (Hospital open public park)	S, Q	12 weeks	60 min	3 times/week	Blank control (Routine pharmacological therapy)
Cheng L et al. (2021)	24-form simplified Tai Chi	Outdoor (Residential open square)	S, Q	24 weeks	60 min	3 times/week	Routine lifestyle control (No structured exercise)
Song QH. et al. (2014)	Chen-style 18-form Tai Chi + paroxetine	Outdoor (Residential open square/green space)	S, Q	6.4 weeks	35 min	14 times/week	Active drug therapy control (Paroxetine, 20 mg/day)
Yang XW (2018)	24-form simplified Tai Chi	Outdoor (Medical university sports ground)	S, Q	16 weeks	60–75 min	3 times/week	Active exercise control (Brisk walking)
Deng HM. et al. (2013)	Outdoor Tai Chi + ethnic music	Outdoor (Open outdoor practice setting)	S, Q	12 weeks	30–60 min	∼7 times/week	Active lifestyle control (Post-dinner walking)

S, supervised; Q, group; U, unsupervised; I, individual; S → U, supervised initially then self-practice.

### 3.4 Risk of bias

The risk of bias of the 15 included RCTs was assessed using the Cochrane Risk of Bias 2.0 (RoB 2.0) tool based on the intention-to-treat principle. The detailed results are presented in Figure 2. According to the Cochrane Handbook for Systematic Reviews of Interventions, each domain and the overall risk of bias were classified as low risk, some concerns, or high risk. Among the 15 studies, 4 were judged to have a low overall risk of bias (38, 40, 45, 47); 10 had some concerns (36, 37, 39, 41–44, 46, 48, 49); and 1 was rated as high risk (35). Specifically, in the randomisation process (Domain 1), only 4 studies (38, 40, 45, 47) clearly reported adequate random sequence generation and rigorous allocation concealment procedures, and were therefore rated as “low risk”; the remaining 11 studies were rated as “some concerns” because they only mentioned “random allocation” without providing detailed information on allocation concealment (35–37, 39, 41–44, 46, 48, 49). In the domain of deviations from intended interventions (Domain D2), although Tai Chi interventions inherently preclude blinding of participants and intervention providers, all 15 studies demonstrated high protocol adherence and no inappropriate deviations, and were strictly analysed according to the intention-to-treat (ITT) principle, thus receiving a “low risk” rating. Regarding missing outcome data (Domain D3), one study (35) was judged to be at high risk because of substantial loss to follow-up without appropriate statistical handling of missing data, while one study (42) was rated as having some concerns because of minor attrition. The remaining 13 studies (36–41, 43–49) were considered to be at low risk. In addition, for outcome measurement (Domain D4), 13 studies (35, 37, 38, 40–49) were rated as low risk because validated psychological assessment instruments were used. Two studies (36, 39) were judged as having some concerns owing to insufficient information regarding assessor blinding or the outcome assessment procedures. Finally, in the domain of selective reporting (Domain D5), 13 studies (35, 37, 38, 40–49) were rated as low risk, as the reported analyses were consistent with the prespecified outcomes. Two studies (36, 39) were judged as having some concerns because no clinical trial registration or detailed statistical analysis plan was available. Overall, although one study was judged to have a high risk of bias because of incomplete outcome data and several studies lacked sufficient information regarding allocation concealment, the included evidence demonstrated generally satisfactory methodological quality, particularly with respect to intervention implementation and outcome reporting.

**Figure 2:**
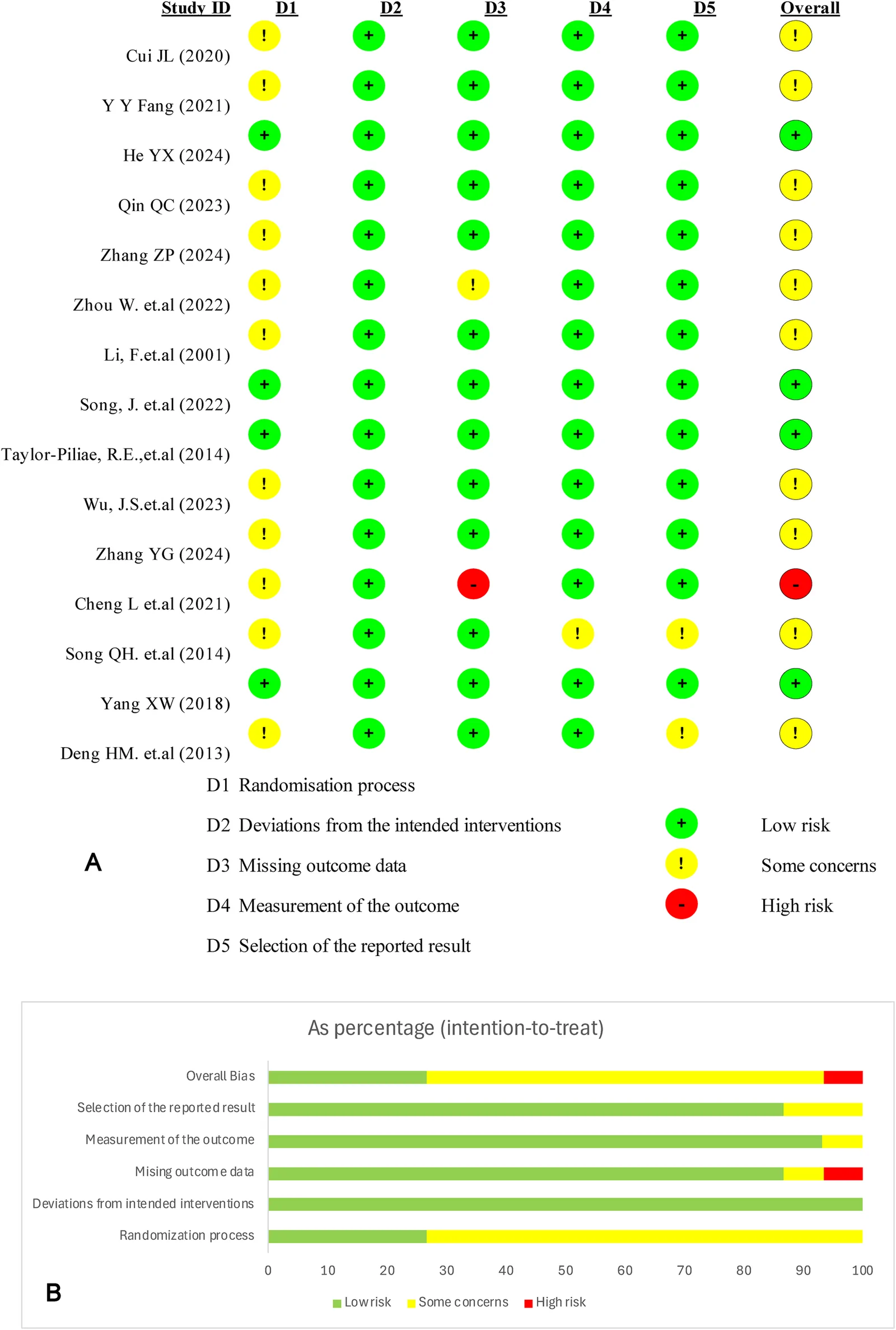
**(A)** Risk of bias ratings. **(B)** Risk of bias graph.

### 3.5 Overall intervention effect

#### 3.5.1 Exercise group and control group

A total of 15 studies were included in this analysis. Given the high heterogeneity among studies (*I*^*2*^ > 50%), a random-effects model and the standardised mean difference (SMD) were used to examine the effects of Tai Chi on depression and anxiety. First, regarding the overall intervention effect on depressive symptoms, 13 studies reported improvements in depression outcomes following Tai Chi intervention (35–38, 40–47, 49). The pooled result from the random-effects model showed that Tai Chi significantly alleviated depressive mood in participants, with a combined effect size of SMD = −0.93 (95% CI: −1.25 to −0.61), and the statistical test indicated a highly significant difference between groups. Although there was substantial heterogeneity across studies (*I*^2^ = 75.1%), examination of individual study effect sizes revealed that, with the exception of one study (40) showing a slight negative trend and two studies (35, 49) reporting non-significant results, the vast majority of independent trials strongly supported the clinical efficacy of Tai Chi for depressive symptoms. Second, for the overall intervention effect on anxiety symptoms, 11 studies assessed the impact of Tai Chi on anxiety levels (36, 38, 39, 41, 42, 44–49). The meta-analytic pooled estimate indicated that, compared with control groups, Tai Chi practice also significantly reduced anxiety scores, with a highly statistically significant combined effect size (SMD = −0.93, 95% CI: −1.23 to −0.64, *I*^*2*^ = 62.9%). The heterogeneity test showed moderate heterogeneity among studies (*I*^2^ = 62.9%). At the individual study level, except for two studies (44, 45) whose confidence intervals crossed the null line (zero), the remaining nine studies consistently demonstrated significant beneficial effects of Tai Chi on anxiety (36, 38, 39, 41, 42, 46–49). In summary, the quantitative data synthesis confirms that Tai Chi exerts clear and considerable overall benefits for both depression and anxiety. It is worth noting that a few individual studies had effect sizes close to zero or confidence intervals containing zero, which may be attributable to small sample sizes, short intervention durations, or the use of active control interventions with equivalent exercise volume (e.g., brisk walking) that might have masked the net effect of Tai Chi (Figures 3, 4).

**Figure 3:**
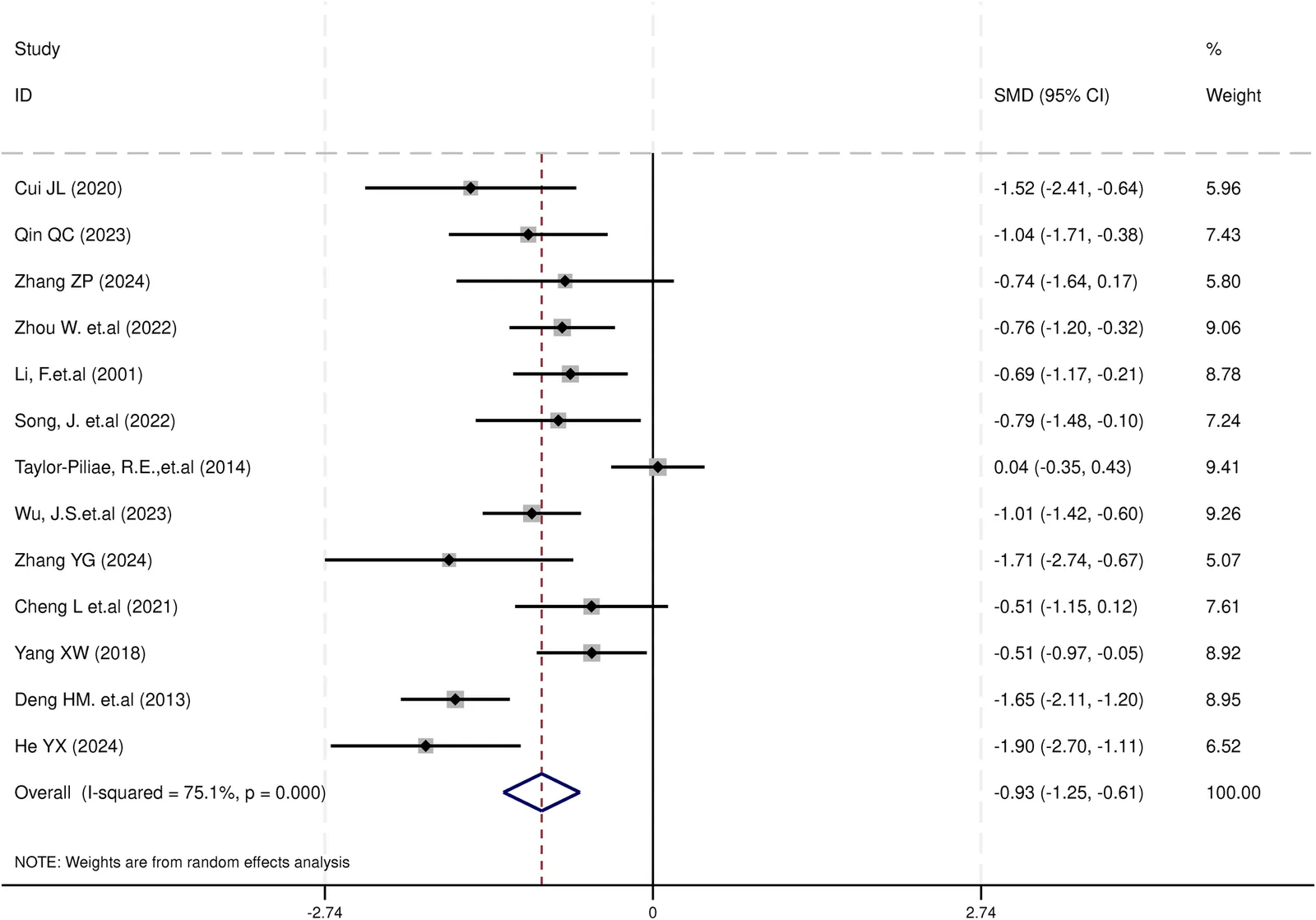
Forest plot of the effect of Tai Chi intervention on depressive symptoms. In the figure, grey squares represent the effect size (standardised mean difference, SMD) for each of the 13 included studies, with the area of the square proportional to the weight of that study in the meta-analysis. Horizontal lines extending from each square indicate the 95% confidence interval of the estimated effect size for that study. The diamond at the bottom represents the pooled effect size derived from the random-effects model, with its width indicating the 95% confidence interval. The vertical dashed line at zero indicates the point of no effect (SMD = 0). Negative effect sizes (i.e., those to the left of the null line) favour Tai Chi intervention (i.e., lower depression scores in the Tai Chi group compared with the control group). The *I*^2^ statistic quantifies the proportion of total variation attributable to between-study heterogeneity.

**Figure 4:**
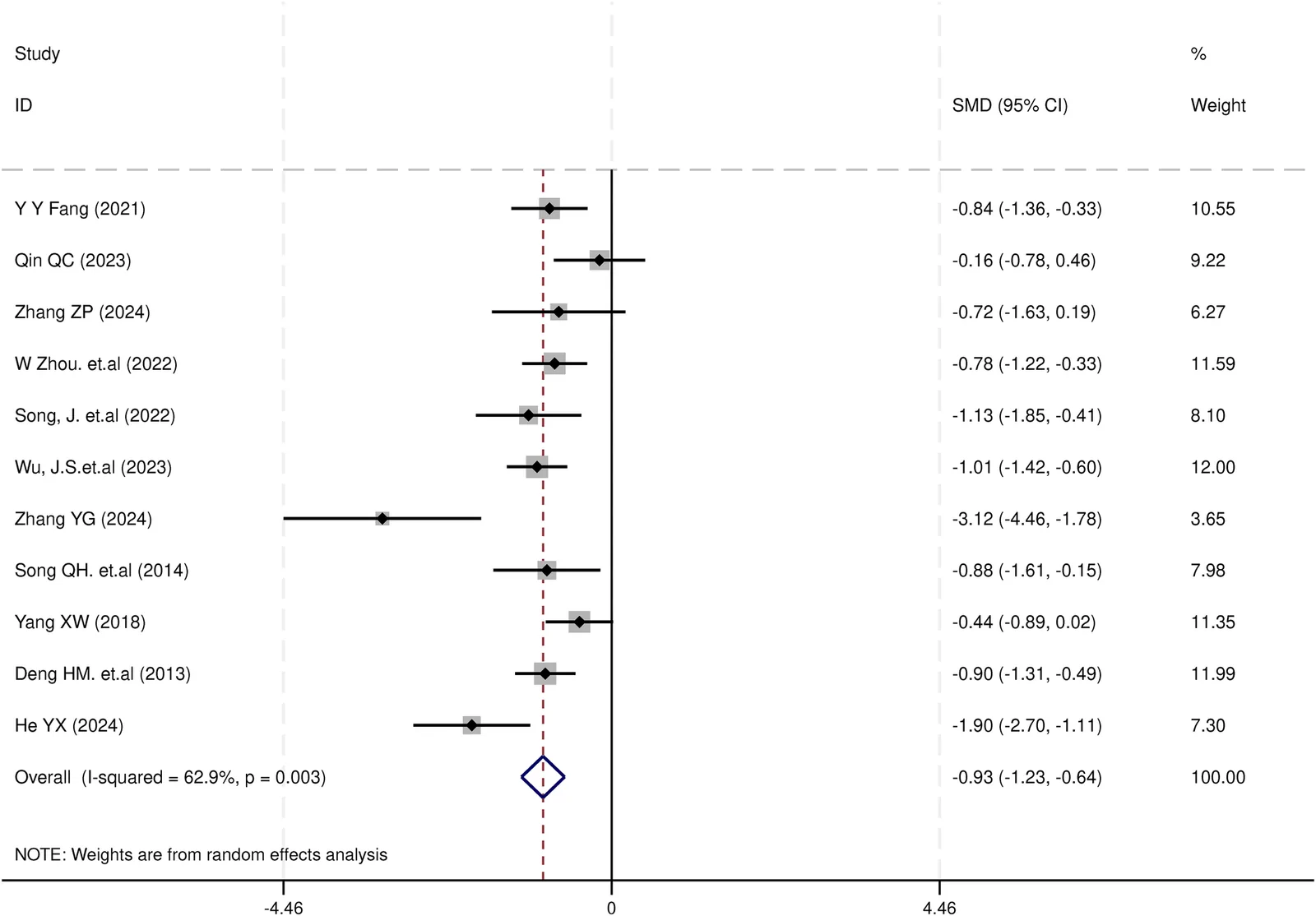
Forest plot of the effect of Tai Chi intervention on anxiety symptoms. In the figure, grey squares represent the effect size (standardised mean difference, SMD) for each of the 11 included studies, with the area of the square proportional to the weight of that study in the meta-analysis. Horizontal lines extending from each square indicate the 95% confidence interval of the estimated effect size for that study. The diamond at the bottom represents the pooled effect size derived from the random-effects model, with its width indicating the 95% confidence interval. The vertical dashed line at zero indicates the point of no effect (SMD = 0). Negative effect sizes (i.e., those to the left of the null line) favour Tai Chi intervention (i.e., lower anxiety scores in the Tai Chi group compared with the control group). The *I*^2^ statistic quantifies the proportion of total variation attributable to between-study heterogeneity.

#### 3.5.2 Regression analysis

Given that covariates such as total intervention duration (weeks), weekly frequency, session duration, exercise environment, and participant age might influence the effects of Tai Chi on improving depression and anxiety, meta-regression analyses were conducted to explore whether these factors served as potential moderators contributing to effect size heterogeneity. The results of the covariate regression analyses are summarized in Tables 3 and 4. For depressive symptoms, none of the examined covariates significantly moderated the pooled effect size. Specifically, intervention duration was not significantly associated with the effect of Tai Chi on depression (*β* = 0.13, 95% CI: −2.17 to 2.43, *p* = 0.90). Similarly, training frequency (*β* = −0.15, 95% CI: −2.07 to 1.77, *p* = 0.86), session duration (*β* = 0.10, 95% CI: −0.97 to 1.17, *p* = 0.83), exercise environment, the primary variable of interest in this study (*β* = −0.14, 95% CI: −2.57 to 2.30, *p* = 0.90), and participants’ age (*β* = −0.11, 95% CI: −0.89 to 0.67, *p* = 0.75) were likewise not significantly associated with the magnitude of improvement in depressive symptoms. For anxiety symptoms, the meta-regression model also indicated that intervention duration (*β* = −1.35, 95% CI: −3.12 to 0.43, *p* = 0.11), training frequency (*β* = 0.10, 95% CI: −0.57 to 0.76, *p* = 0.73), session duration (*β* = −0.20, 95% CI: −1.76 to 1.37, *p* = 0.76), and exercise environment (*β* = 0.82, 95% CI: −0.65 to 2.29, *p* = 0.21) did not independently moderate the effects of Tai Chi on anxiety. Notably, participants' age showed a marginally significant moderating trend (*β* = 1.35, 95% CI: −0.29 to 3.00, *p* = 0.09), suggesting that older participants may exhibit a different trajectory of anxiety improvement following Tai Chi intervention. However, this association did not reach the conventional threshold for statistical significance.

**Table 3:** Meta-regression analysis of Tai Chi intervention on depressive symptoms.

_ES	Coefficient	Std. err.	*t*	*P*>|*t*|	[95% conf. interval]
Duration(weeks)	0.13	0.97	0.13	0.90	−2.17 to 2.43
frequency(week)	−0.15	0.81	−0.18	0.86	−2.07 to 1.77
Time(min)	0.10	0.45	0.22	0.83	−0.97 to 1.17
Exercise environment	−0.14	1.03	−0.13	0.90	−2.57 to 2.30
Age	−0.11	0.33	−0.34	0.75	−0.89 to 0.67
_cons	−0.69	2.47	−0.28	0.79	−6.53 to 5.16

ES, effect size (used in original table header, clarified as SMD); Std. err., standard error; CI, confidence interval.

**Table 4:** Meta-regression analysis of Tai Chi intervention on anxiety symptoms.

_ES	Coefficient	Std. err.	*t*	*P*>|*t*|	[95% conf. interval]
Duration(weeks)	−1.35	0.69	−1.95	0.11	−3.12 to 0.43
frequency(week)	0.10	0.26	0.37	0.73	−0.57 to 0.76
Time(min)	−0.20	0.61	−0.32	0.76	−1.76 to 1.37
Exercise environment	0.82	0.57	1.43	0.21	−0.65 to 2.29
Age	1.35	0.64	2.12	0.09	−0.29 to 3.00
_cons	−3.43	1.76	−1.95	0.11	−7.95 to 1.10

#### 3.5.3 Subgroup and sensitivity analyses

To further explore potential sources of between-study heterogeneity, subgroup analyses were performed using a random-effects model according to intervention duration, training frequency, session duration, exercise environment, and participants' age. The results for depression and anxiety are presented in Tables 5, 6. Interventions lasting 11–15 weeks yielded larger effect sizes for both depression (SMD = −1.05) and anxiety (SMD = −1.07), whereas effect sizes decreased for interventions extending beyond 15 weeks (SMD = −0.70 and −0.44, respectively), suggesting that prolonging intervention duration did not incrementally enhance mental health benefits. Regarding session duration, the ≤30 min subgroup demonstrated larger improvements in both depression (SMD = −1.28) and anxiety (SMD = −1.29); however, as this subgroup included only 2 RCTs, these findings warrant cautious interpretation. In terms of practice frequency, practice at >3 sessions per week yielded a slightly higher effect size for depression (SMD = −1.20), whereas 3 sessions per week showed the largest effect size for anxiety (SMD = −1.06). Overall, no clear dose–response relationship was observed across the training parameters. Regarding the exercise environment, descriptively larger effect sizes were observed in outdoor practice settings compared with indoor settings for both depression (SMD = −1.06, 95% CI: −1.75 to −0.36 vs. SMD = −0.88, 95% CI: −1.24 to −0.51) and anxiety (SMD = −1.08, 95% CI: −1.76 to −0.41 vs. SMD = −0.90, 95% CI: −1.23 to −0.58). However, the outdoor subgroup comprised only 4 studies for each outcome and exhibited substantial between-study heterogeneity (depression: *I*^2^ = 81.5%; anxiety: *I*^2^ = 78.7%). More importantly, consistent with the multivariable meta-regression findings (Tables 3, 4), this descriptive difference did not translate into a statistically significant moderating effect. Given that several subgroups (e.g., outdoor setting, ≤30 min duration, >15 weeks duration) contained limited studies and high residual heterogeneity, all subgroup findings should be interpreted as exploratory and hypothesis-generating rather than definitive evidence for specific moderating effects or optimal prescriptions. An exploratory geographical subgroup analysis for depressive symptoms showed a larger pooled effect among studies conducted in China than among the two studies conducted in the United States; however, the between-subgroup difference did not reach statistical significance (*P* = 0.059; Supplementary Figure S1). Given the limited number of US studies, these findings should be interpreted cautiously. A geographical subgroup analysis was not performed for anxiety because all contributing studies were conducted in China.

**Table 5:** Subgroup analysis of Tai Chi intervention on depressive symptoms.

Group	Sub-Group	*K*	*N*	SMD	95% CI	*p*	*I* ^2^
Duration(weeks)	≤10	1	85	−0.76	−1.20 to −0.32	.	.%
	11 ≤ 15	8	461	−1.05	−1.56 to −0.55	0.000	83.1%
	＞15	4	206	−0.70	−1.06 to −0.33	0.211	33.5%
Frequency(week)	＜3	2	92	−0.70	−1.12 to −0.28	0.933	0.00%
	3	9	475	−0.92	−1.33 to −0.50	0.000	76.3%
	＞3	2	185	−1.20	−2.08 to −0.33	0.006	86.9%
Time(min)	≤30	2	121	−1.28	−2.40 to −0.17	0.014	83.5%
	31 ≤ 60	9	530	−0.87	−1.28 to −0.46	0.000	78.2%
	＞60	2	101	−0.95	−1.93 to 0.04	0.046	74.9%
Exercise environment	Indoor	9	518	−0.88	−1.24 to −0.51	0.000	72.6%
	Outdoor	4	234	−1.06	−1.75 to −0.36	0.001	81.5%
Age	≤30	3	214	−0.97	−1.30 to −0.65	0.296	17.9%
	31 ≤ 59	2	121	−0.23	−0.96 to 0.49	0.124	57.7%
	≥60	8	417	−1.05	−1.45 to −0.66	0.002	69.8%

**Table 6:** Subgroup analysis of Tai Chi intervention on anxiety symptoms.

Group	Sub-Group	*K*	*N*	SMD	95% CI	*p*	*I* ^2^
Duration(weeks)	≤10	2	117	−0.80	−1.18 to −0.43	0.810	0.00%
	11 ≤ 15	8	417	−1.07	−1.47 to −0.67	0.002	68.8%
	＞15	1	75	−0.44	−0.89 to 0.22	.	.%
Frequency(week)	＜3	1	20	−0.72	−1.63 to −0.19	.	.%
	3	7	372	−1.06	−1.55 to −0.57	0.000	77.5%
	＞3	3	217	−0.85	−1.13 to −0.57	0.915	0.00%
Time(min)	≤30	2	121	−1.29	−2.39 to −0.19	0.015	83.0%
	31 ≤ 60	8	413	−0.94	−1.28 to −0.59	0.019	58.2%
	＞60	1	75	−0.44	−1.23 to 0.02	.	.%
Exercise environment	Indoor	7	382	−0.90	−1.23 to −0.58	0.048	52.7%
	Outdoor	4	227	−1.08	−1.76 to −0.41	0.003	78.7%
Age	≤30	2	188	−0.90	−1.20 to −0.60	0.439	0.00%
	31 ≤ 59	2	40	−1.87	−4.22 to 0.48	0.004	88.1%
	≥60	7	381	−0.84	−1.19 to −0.50	0.021	59.9%

As a supplementary sensitivity analysis, the primary meta-analyses were repeated after excluding the seven graduate theses/dissertations and retaining only studies published in peer-reviewed journals. The pooled effect on depressive symptoms remained statistically significant (*k* = 7; SMD = −0.77, 95% CI: −1.20 to −0.34; *I*^*2*^ = 81.8%), consistent with the direction and significance of the primary analysis. Similarly, the pooled effect on anxiety symptoms remained significant (*k* = 5; SMD = −0.92, 95% CI: −1.14 to −0.70; *I*^*2*^ = 0.0%). These findings indicate that the main results were robust to the exclusion of dissertation-based studies (Supplementary Figures S2, S3).

## 4 Discussion

This study conducted a systematic review and meta-analysis of 15 RCTs encompassing 847 participants to systematically evaluate the effects of Tai Chi on depressive and anxiety symptoms, and further explored the potential moderating role of the physical exercise environment. The results demonstrated that Tai Chi significantly improved both depressive (SMD = −0.93) and anxiety symptoms (SMD = −0.93), indicating that as a mind-body exercise, it confers stable and substantial intervention value for mental health promotion. These findings are generally consistent with those of several recent systematic reviews and meta-analyses. For instance, Lu et al.'s umbrella review indicated that among various exercise modalities, Tai Chi and traditional Qigong exhibited relatively high overall efficacy for psychological disorders (50). Park et al. further confirmed that Tai Chi and Qigong interventions significantly improved depression, anxiety, and overall psychiatric symptoms in individuals with mental illness (51). Moreover, Tai Chi has demonstrated stable antidepressant effects both in older adults with depression (52) and in adolescents and young adults with depressive symptoms (53). The present study, based on a broader scope of included participants, disease types, and intervention protocols, reaffirms these conclusions and further enriches the evidence base for Tai Chi as a non-pharmacological strategy for alleviating negative emotions.

Regarding the exercise environment, our multivariable meta-regression analyses indicated that practice setting was not a statistically significant independent moderator for improvements in either depression (*p* = 0.90) or anxiety (*p* = 0.21) after adjusting for potential confounders such as age and intervention duration. Although univariate subgroup analyses descriptively showed larger effect sizes for outdoor settings compared with indoor settings (depression: SMD = −1.06 vs. −0.88; anxiety: SMD = −1.08 vs. −0.90), the outdoor subgroup was limited to only 4 studies per outcome and exhibited substantial between-study heterogeneity (*I*^2^ > 78%). Therefore, the available statistical evidence does not support an independent moderating effect of the practice setting, and these numerical variations should be interpreted cautiously as preliminary and hypothesis-generating rather than confirmatory.

From a theoretical perspective, Stress Recovery Theory and Attention Restoration Theory suggest that exposure to natural elements can facilitate psychological restoration and alleviate negative affect (54, 55), which aligns with concepts in green exercise research (56–58). Mind–body practices like Tai Chi might theoretically interact synergistically with restorative outdoor environments to enhance emotional recovery (59–61). However, our multivariable meta-regression did not identify a statistically significant independent moderating effect of the practice setting. Several potential explanations may account for this finding. First, from an environmental perspective, real-world outdoor practice inevitably involves uncontrolled contextual variables—such as weather fluctuations, ambient noise, and social evaluative concerns in public spaces (62–65)-which may have confounded potential environmental benefits. Second, from an intervention perspective, although outdoor settings historically represent the traditional baseline for Tai Chi, modern adaptations delivered indoors still retain the core therapeutic components of mindful movement, breath regulation, and attentional focus. The substantial effect sizes observed within the indoor subgroup (SMD = −0.88 for depression, SMD = −0.90 for anxiety) provide encouraging empirical support for the pragmatic value of indoor Tai Chi, particularly in clinical and institutional settings (e.g., hospital wards, nursing homes, and rehabilitation facilities) where mobility-impaired or frail individuals face barriers to outdoor activity. Nevertheless, given the small number of outdoor trials (*k* = 4 per outcome) and substantial between-study heterogeneity, current evidence cannot definitively confirm or exclude a moderating effect of the environment, nor does it establish absolute equivalence between settings. Rigorously controlled, head-to-head RCTs with standardized environmental exposure assessments are warranted to directly evaluate the relative efficacy and context-specific mechanisms of indoor vs. outdoor Tai Chi interventions.

Beyond environmental factors, this study further explored intervention dosage. Subgroup analysis revealed that Tai Chi protocols lasting 11–15 weeks, with a frequency of ≥3 sessions per week and a session duration of ≤30 min yielded relatively large mental health benefits. This finding is broadly consistent with a recent meta-analysis on exercise interventions for depression prevention, which indicated that moderate intervention durations and single-session exercise lasting approximately 30–40 min produce favourable mental health benefits (66). A higher practice frequency combined with moderate session duration may be more conducive to maintaining long-term exercise adherence, thereby enhancing intervention effects—and adherence to mind-body exercise itself is an important factor influencing mental health gains (67). Furthermore, higher-frequency exercise may further enhance mental health benefits through sustained activation of the stress-buffering system and reduction of cortisol levels (68). Notably, the subgroup with the longest intervention duration (>15 weeks) did not produce the largest effect sizes; rather, the numerical values were lower than those of the 11–15-week group. This phenomenon is not uncommon in psychological and behavioural intervention research. From the perspective of behavioural maintenance, the effectiveness of long-term exercise interventions depends not only on the exercise prescription itself but also on multiple factors including individual motivation, self-efficacy, and the capacity for behavioural maintenance. As intervention duration extends, participants' exercise adherence may gradually decline, thereby attenuating intervention effects (69). For Tai Chi specifically, once the movement sequences reach a plateau in the learning curve, the absence of progressive variation may fail to sustain practitioners' cognitive engagement and emotional arousal. In addition, in age-based subgroup analysis, this study found that both young adults (≤30 years) and older adults (≥60 years) exhibited relatively stable mental health improvements, whereas the 31–59-year age group did not reach statistical significance. This result may relate to the higher levels of occupational stress, family responsibilities, and barriers to exercise participation commonly faced during midlife. Previous systematic reviews have indicated that time constraints, work commitments, and family responsibilities are major barriers to sustained physical activity participation in middle-aged populations, which may in turn affect intervention adherence and long-term outcomes (70). However, given the uneven distribution of studies across age subgroups in this analysis, the above findings require further validation through additional RCTs targeting specific age groups. Additionally, the diversity of Tai Chi styles and routine forms across the included studies (ranging from the simplified 24-form Yang-style, 8-form, 16-form, and seated Tai Chi to dynamic Chen-style Tai Chi) represents another potential source of heterogeneity; differences in movement complexity, cognitive load, and energy expenditure across styles may engage distinct emotional regulation pathways, yet due to the imbalanced distribution of the sample (with Yang-style derivatives predominating), the present study did not quantitatively examine style as an independent moderating variable. Future research employing standardized classifications and head-to-head comparisons of specific Tai Chi styles will help clarify whether particular routine forms offer superior emotional benefits for specific clinical populations.

In addition to stylistic and demographic variations, geographical and sociocultural context represents another potential source of heterogeneity. In our exploratory analysis, although the pooled effect estimate was numerically larger among studies conducted in China than among those conducted in the United States (SMD = −1.06 vs. −0.31), the between-subgroup difference did not reach statistical significance (*P* = 0.059). Importantly, only two US trials were available, and their pooled estimate was imprecise with substantial heterogeneity (*I*^2^ = 81.4%). Therefore, the apparent geographical difference should not be interpreted as evidence that Tai Chi is more effective in China than in the United States. Moreover, both US trials were conducted in indoor settings, making geographical location partially confounded with the exercise-environment characteristic examined in the present review. Differences in participant characteristics, cultural familiarity with Tai Chi, intervention protocols, control conditions, and other study-level characteristics may also contribute to the observed variation. Larger numbers of RCTs conducted outside China are therefore needed before geography can be reliably evaluated as an independent moderator.

In summary, the present findings robustly confirm the intervention value of Tai Chi for alleviating depression and anxiety, while demonstrating that its mental health benefits possess broad generalizability across diverse practice environments. Rather than presuming an inherent superiority of outdoor settings, future research should implement methodologically rigorous, head-to-head RCTs with standardized environmental exposure assessments to elucidate whether and how specific environmental attributes optimize the effects of mind-body exercise interventions.

## 5 Limitations of the study

Although this systematic review and meta-analysis has comprehensively evaluated the effects of Tai Chi on depressive and anxiety symptoms and explored the potential moderating role of indoor vs. outdoor practice settings from an environmental perspective, several limitations should be acknowledged.

First, the geographic distribution of the included studies is somewhat limited. Among the 15 RCTs included in this review, 13 were conducted in China and only 2 in the United States, and all studies reporting anxiety outcomes were conducted exclusively in China. Although our exploratory subgroup analysis for depressive symptoms did not yield a statistically significant difference between Chinese and US trials (*P* = 0.059), the small number of trials outside China resulted in wide confidence intervals and limited precision. As a mind–body exercise originating from traditional Chinese culture, Tai Chi may be perceived, practiced, and contextually delivered differently across countries and cultural settings. Therefore, the cross-cultural generalizability of our findings should be interpreted with caution. Future studies should incorporate more high-quality RCTs conducted across diverse international regions to enhance the external validity of the conclusions.

Second, the environmental subgroup analysis in the present study has several methodological limitations. Practice settings were classified as either indoor or outdoor based on descriptions provided in the original studies. However, in most of the included trials, the exercise environment was not incorporated as a primary design factor but rather reflected the actual location where the intervention was delivered. Consequently, the environmental subgroup analysis is more likely to capture potential differences in the effectiveness of Tai Chi across practice settings than to establish a causal relationship between the exercise environment and improvements in mental health. Furthermore, descriptions of environmental characteristics in the original studies were generally limited. Most studies did not distinguish among different types of natural environments, such as parks, forests, or community green spaces, nor did they provide standardized assessments of objective environmental exposure indicators, including green visibility, air quality, temperature, humidity, and ambient noise. Therefore, it remains unclear whether and to what extent specific environmental characteristics might influence the mental health benefits of Tai Chi.

Third, long-term follow-up data remain scarce in the existing literature. Most of the RCTs included in this review primarily evaluated the immediate effects of Tai Chi at the end of the intervention, with few providing follow-up data beyond 3 months. Consequently, the present study was unable to assess the long-term sustainability of Tai Chi's beneficial effects on depression and anxiety, nor could it determine whether the psychological gains associated with different practice settings are maintained over time.

Finally, there remains considerable heterogeneity across the included studies in terms of participant characteristics, clinical health status, intervention duration, training dosage, and outcome assessment tools. In particular, although multiple Tai Chi styles (e.g., Yang-style, Chen-style, seated, and simplified forms) were represented, the overwhelming predominance of Yang-style variations and the presence of only a single trial for Chen-style or music-accompanied Tai Chi precluded robust subgroup analyses or meta-regressions based on style categories due to severe category imbalance and limited statistical power. Consequently, style-specific variations remain an unexplored source of clinical heterogeneity in the present synthesis. Although we employed a random-effects model and conducted subgroup analyses across available parameters, residual heterogeneity cannot be completely ruled out.

Future research should conduct more large-sample, multicentre, and methodologically rigorous RCTs that incorporate the exercise environment and specific Tai Chi styles as independent study factors into the research design, establish standardized environmental exposure assessment systems, and integrate objective environmental indicators with neurophysiological measures. Such efforts would help clarify whether different practice settings exert a true moderating effect on Tai Chi's mental health outcomes, thereby providing robust evidence for optimizing context-tailored mind–body interventions.

## 6 Conclusions

This systematic review and meta-analysis demonstrates that Tai Chi significantly improves depressive and anxiety symptoms in adults, confirming its value as an effective non-pharmacological mind–body intervention for mental health promotion. Although univariate subgroup analyses descriptively observed larger effect sizes in outdoor settings than in indoor settings, multivariable meta-regression analyses indicated that the exercise environment was not a statistically significant independent moderator of mental health outcomes. Given the limited number of outdoor trials and substantial between-study heterogeneity, current empirical evidence does not justify a presumed superiority of outdoor practice over indoor practice. Rather, the consistent improvements observed across diverse settings indicate that the psychological benefits of Tai Chi are robust and not strictly bounded by the physical practice environment, supporting its pragmatic implementation across institutional, clinical, and community settings. In addition, intervention protocols lasting 11–15 weeks, with a frequency of ≥3 sessions per week and a session duration of ≤30 min, demonstrated relatively superior mental health benefits. Future research should prioritize large-sample, multicentre, and methodologically rigorous RCTs that incorporate the exercise environment as an independent factor in the study design and employ standardized environmental exposure assessments to determine whether and under what conditions specific environmental contexts provide incremental benefits for mind–body interventions.
