# Supplementary material for: Does exercise environment moderate the effects of Tai Chi on depression and anxiety? A systematic review and meta-analysis of indoor vs. outdoor practice settings
Source: Front Sports Act Living. 2026 Sep 29;8:1955833. doi: 10.3389/fspor.2026.1955833 (PMC13660419; doi:10.3389/fspor.2026.1955833)

Supplementary Material

Manuscript ID: 1955833

Title: Does Exercise Environment Moderate the Effects of Tai Chi on Depression and Anxiety? A Systematic Review and Meta-Analysis of Indoor Versus Outdoor Practice Settings

Figure S1. Forest plot of exploratory subgroup analysis for depressive symptoms stratified by study location (Subgroup 1: China; Subgroup 2: United States).

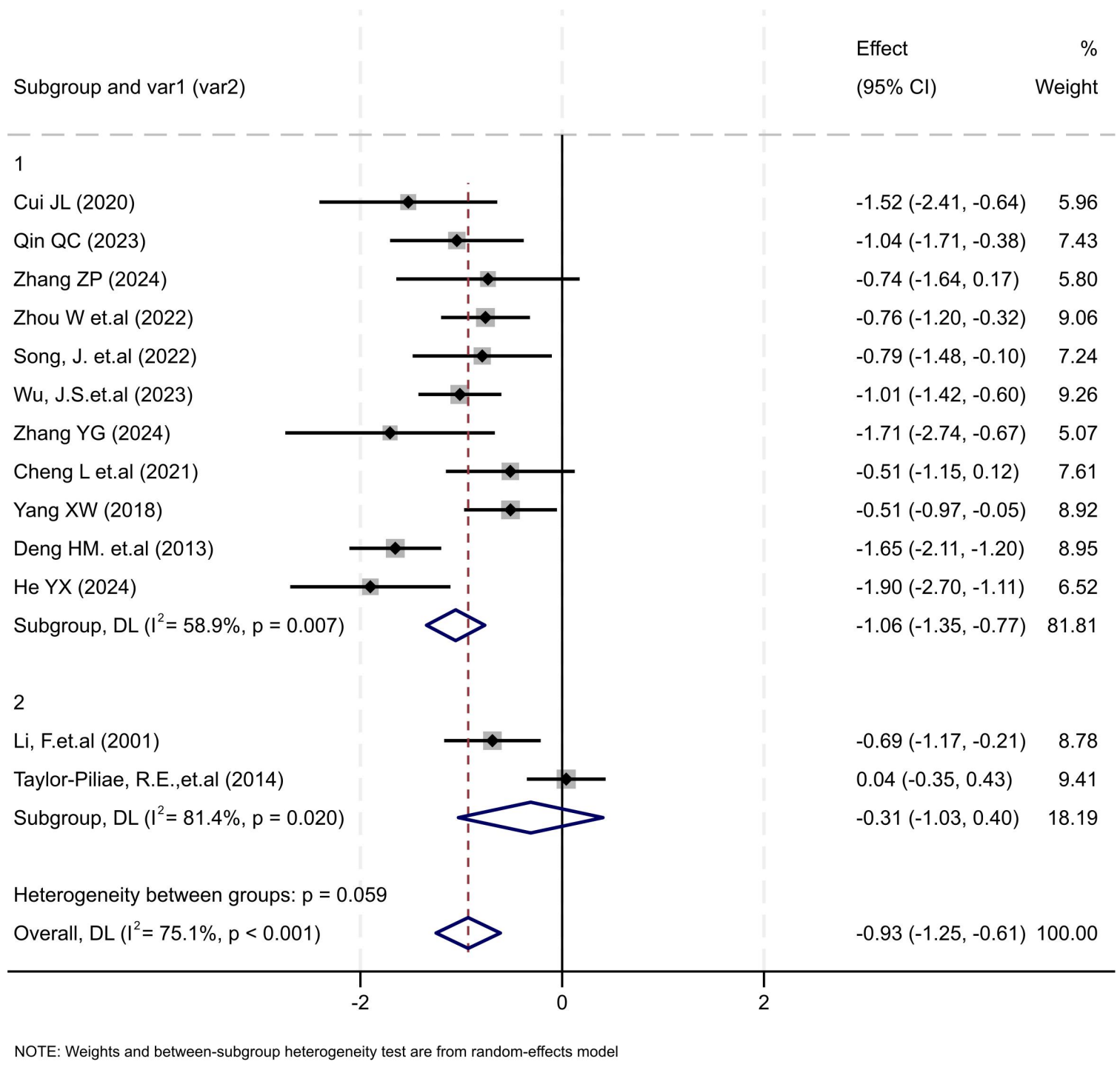

NOTE: Weights and between-subgroup heterogeneity test are derived from the random-effects model. SMD: standardized mean difference; CI: confidence interval. Subgroup 1 = studies conducted in China; Subgroup 2 = studies conducted in the United States.

Figure S2. Sensitivity analysis of the effect of Tai Chi on depressive symptoms after excluding graduate theses/dissertations.

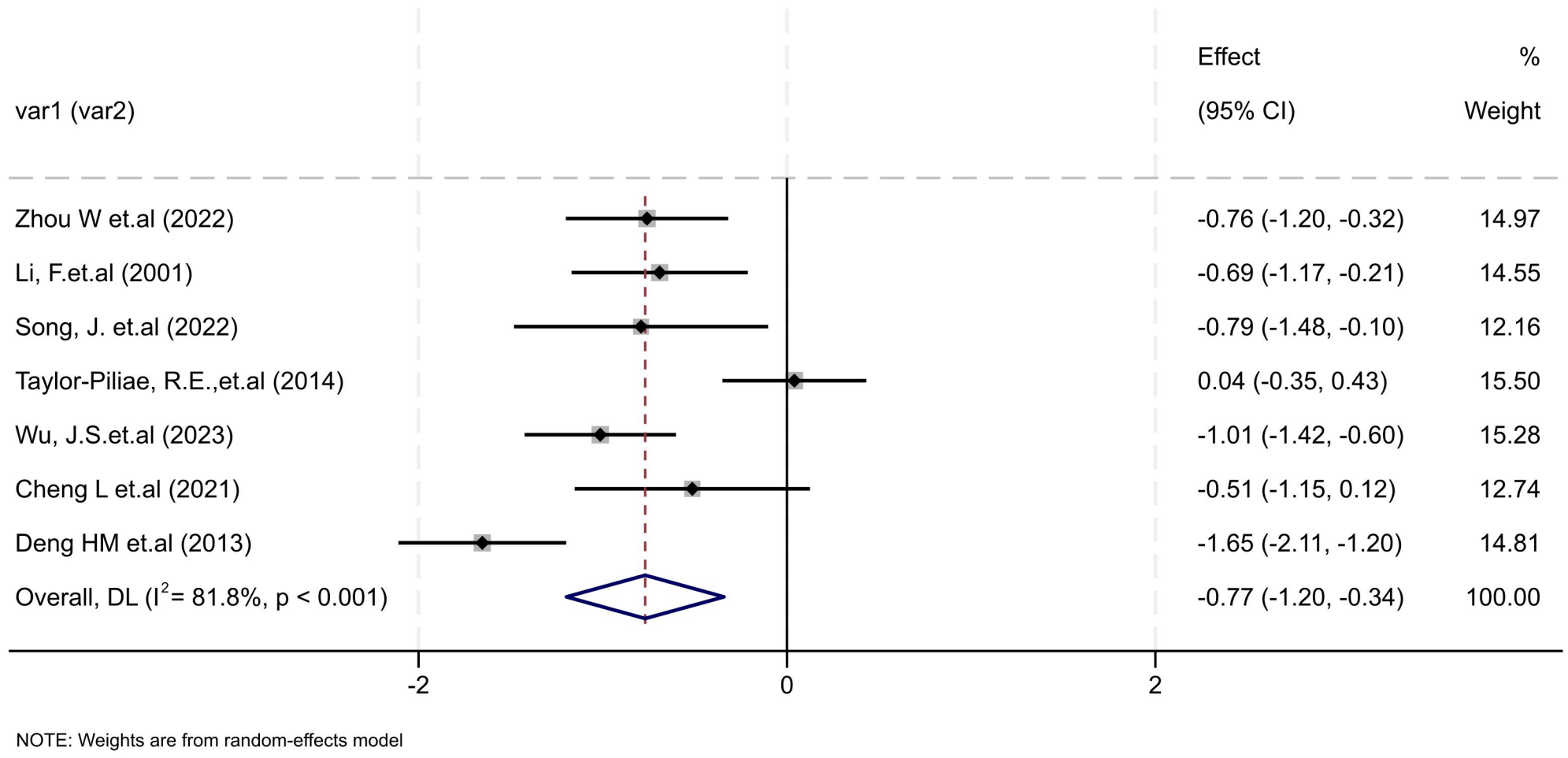

Figure S3. Sensitivity analysis of the effect of Tai Chi on anxiety symptoms after excluding graduate theses/dissertations.

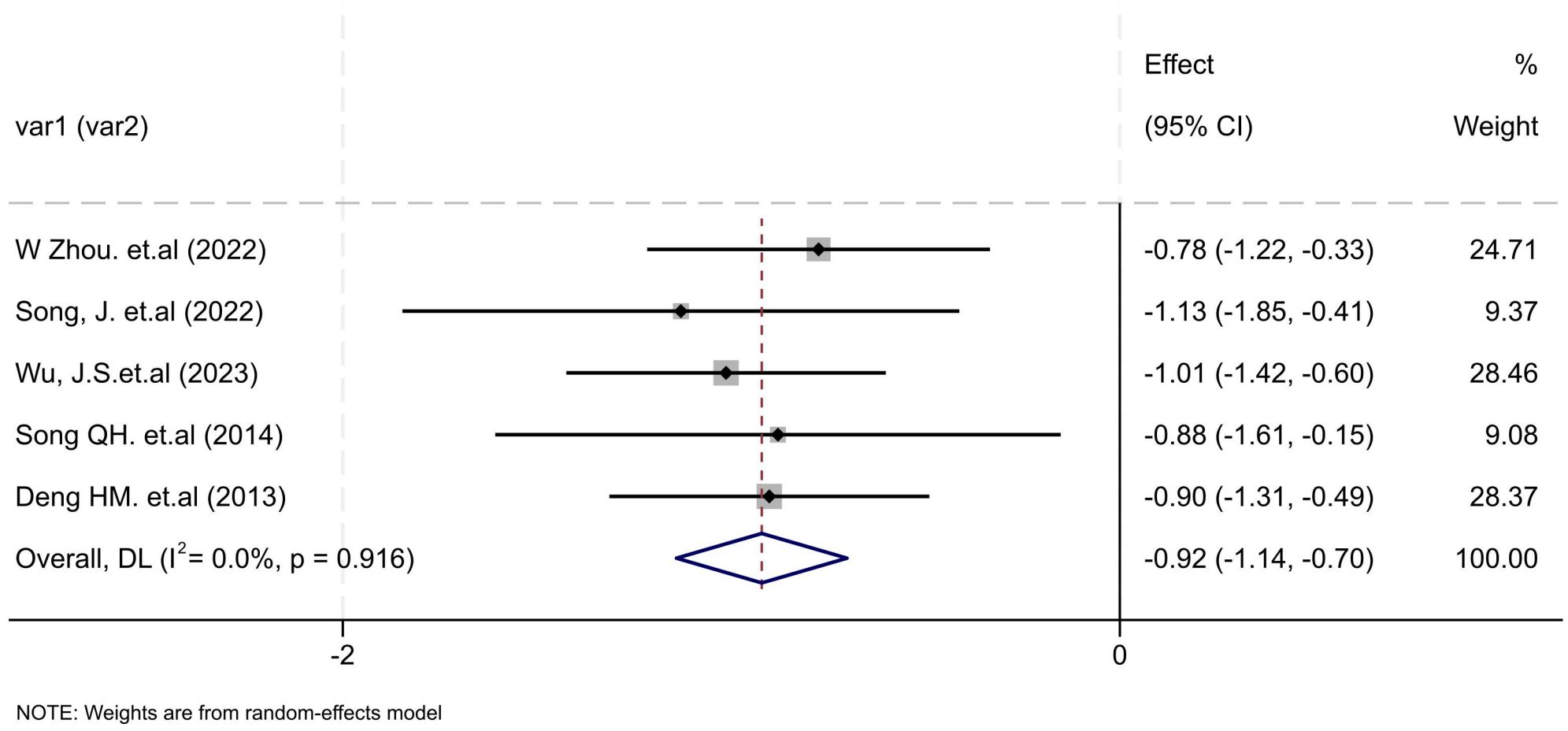

Supplement: Supplementary file 1 [file Datasheet1.pdf]
